# Vessel architecture imaging using multiband gradient-echo/spin-echo EPI

**DOI:** 10.1371/journal.pone.0220939

**Published:** 2019-08-09

**Authors:** Ke Zhang, Seong Dae Yun, Simon M. F. Triphan, Volker J. Sturm, Lukas R. Buschle, Artur Hahn, Sabine Heiland, Martin Bendszus, Heinz-Peter Schlemmer, N. Jon Shah, Christian H. Ziener, Felix T. Kurz

**Affiliations:** 1 Department of Radiology, German Cancer Research Center, Heidelberg, Germany; 2 Department of Neuroradiology, Heidelberg University Hospital, Heidelberg, Germany; 3 Institute of Neuroscience and Medicine– 4, Medical Imaging Physics, Forschungszentrum Jülich, Germany; 4 Department of Diagnostic & Interventional Radiology, Heidelberg University Hospital, Heidelberg, Germany; 5 Department of Physics and Astronomy, University of Heidelberg, Heidelberg, Germany; 6 Department of Neurology, Faculty of Medicine, JARA, RWTH Aachen University, Aachen, Germany; University of Pécs Medical School, HUNGARY

## Abstract

**Objectives:**

To apply the MB (multiband) excitation and blipped-CAIPI (blipped-controlled aliasing in parallel imaging) techniques in a spin and gradient-echo (SAGE) EPI sequence to improve the slice coverage for vessel architecture imaging (VAI).

**Materials and methods:**

Both MB excitation and blipped-CAIPI with in-plane parallel imaging were incorporated into a gradient-echo (GE)/spin-echo (SE) EPI sequence for simultaneous tracking of the dynamic MR signal changes in both GE and SE contrasts after the injection of contrast agent. MB and singleband (SB) excitation were compared using a 20-channel head coil at 3 Tesla, and high-resolution MB VAI could be performed in 32 glioma patients.

**Results:**

Whole-brain covered high resolution VAI can be achieved after applying multiband excitation with a factor of 2 and in-plane parallel imaging with a factor of 3. The quality of the images resulting from MB acceleration was comparable to those from the SB method: images were reconstructed without any loss of spatial resolution or severe distortions. In addition, MB and SB signal-to-noise ratios (SNR) were similar. A relative low g-factor induced from the MB acceleration method was achieved after using a blipped-CAIPI technique (1.35 for GE and 1.33 for SE imaging). Performing quantitative VAI, we found that, among all VAI parametric maps, microvessel type indicator (MTI), distance map (I) and vascular-induced bolus peak-time shift (VIPS) were highly correlated. Likewise, VAI parametric maps of slope, slope length and short axis were highly correlated.

**Conclusions:**

Multiband accelerated SAGE successfully doubles the number of readout slices in the same measurement time when compared to conventional readout sequences. The corresponding VAI parametric maps provide insights into the complexity and heterogeneity of vascular changes in glioma.

## Introduction

Vessel architecture imaging (VAI) MRI is a recently coined term for a technique that noninvasively measures parameters who describe the structural heterogeneity of brain microvasculature [1–4]. The technique uses a temporal shift that occurs between the time-resolved curves of gradient-echo (GE) relaxation rate R_2_^*^ and spin-echo (SE) relaxation rate R_2_ during contrast bolus administration. Depending on the structural properties of the microvessels, as well as their oxygen content and spatial arrangement, the different GE and SE images produce an apparent variation in the respective MRI signal readouts [5–9]. This effect is based on the varying susceptibility differences between blood vessels and surrounding tissue, as well as on their architecture in general, i.e. their structural alignment, density and the characterization of their vessel segment succession from arterial inflow via capillaries to venous outflow. Specifically, local differences in vessel size and bolus transit time between arterial inflow and venous outflow vessels can result in a relative shift in the shape and peak positions of the two relaxation rate curves, an effect first observed and described in [1]. When visualized in a time-parameterized plot, the pairwise GE and SE data points form a vortex or hysteresis curve of a certain shape that depends on the type of vascular architecture. Naturally, the loop direction depends on the axes that define the representation plane; in this study, we always plot GE against SE values to acquire vessel vortex curves. These curves therefore follow either a clockwise or a counterclockwise direction [1, 2]. Furthermore, the shape and enclosed volume of vessel vortex curves depend on the combination of vessel type and caliber. Typically, when the vascular system contains mostly venule-like vessels, the vessel vortices follow a counterclockwise direction. In contrast, if the vascular system contains mostly arteriole-type vessels, the vessel vortices transverse in a clockwise direction [3].

Previous studies found evidence for a dominance of the counterclockwise loop in normal tissue, and a possible alternation of the loop direction in a tumor, indicating a substantial change in tumor vasculature and/or permeability [1]. The size and direction of the hysteresis loop was first analyzed on a voxel basis for a small group in brain tumor (N = 4) and stroke (N = 13) patients, to show that the dominant counterclockwise loop direction found in [1] was a result of averaging of both directions present in the tissue [2]. An observed alternation of the loop direction in tumors was reported as significant, while the one noticeable in the stroke area was not [2]. The method was also applied to a patient cohort with recurrent glioblastoma, where it was found that the alternation of the loop direction in response to therapy predicts a longer patient survival [3]. The latter study used the term “vessel vortex curve” as opposed to “hysteresis loop” [1]; to avoid confusion, we only use the first term in our study. Likewise, the term VAI was first introduced in [3] as a mapping procedure of the vessel vortex curve, while the term vessel-size imaging, first used in [10], was renamed vessel caliber MRI.

VAI was also applied to examine differences in vasculature between different glioma types and to track angiogenesis in glioma growth to provide an imaging-based tumor grade prediction for low and high grade glioma [11].

To obtain VAI parameters, a dual GE/SE EPI sequence is needed to simultaneously track dynamic signal changes in both GE and SE images during contrast bolus administration. It is therefore important to acquire GE and SE signals with a high temporal sampling rate of 1-2s, usually implemented with a short repetition time (TR). Yet, for such short TR’s, it is not possible to cover the whole brain with a combined GE and SE readout. Whole-brain coverage, however, is mandatory for MR examinations in clinical routine. Specifically, brain tumors may appear in two different regions that cannot be covered with one image slab. In addition, reduced coverage has the problem of an inconsistent coverage in a longitudinal study of growing brain tumors. On the other hand, varying coverage becomes an issue in normalization of the perfusion maps to a reference region, as a consistent reference region is more difficult to find, resulting in less repeatable measurements [12].

**Fig 1:**
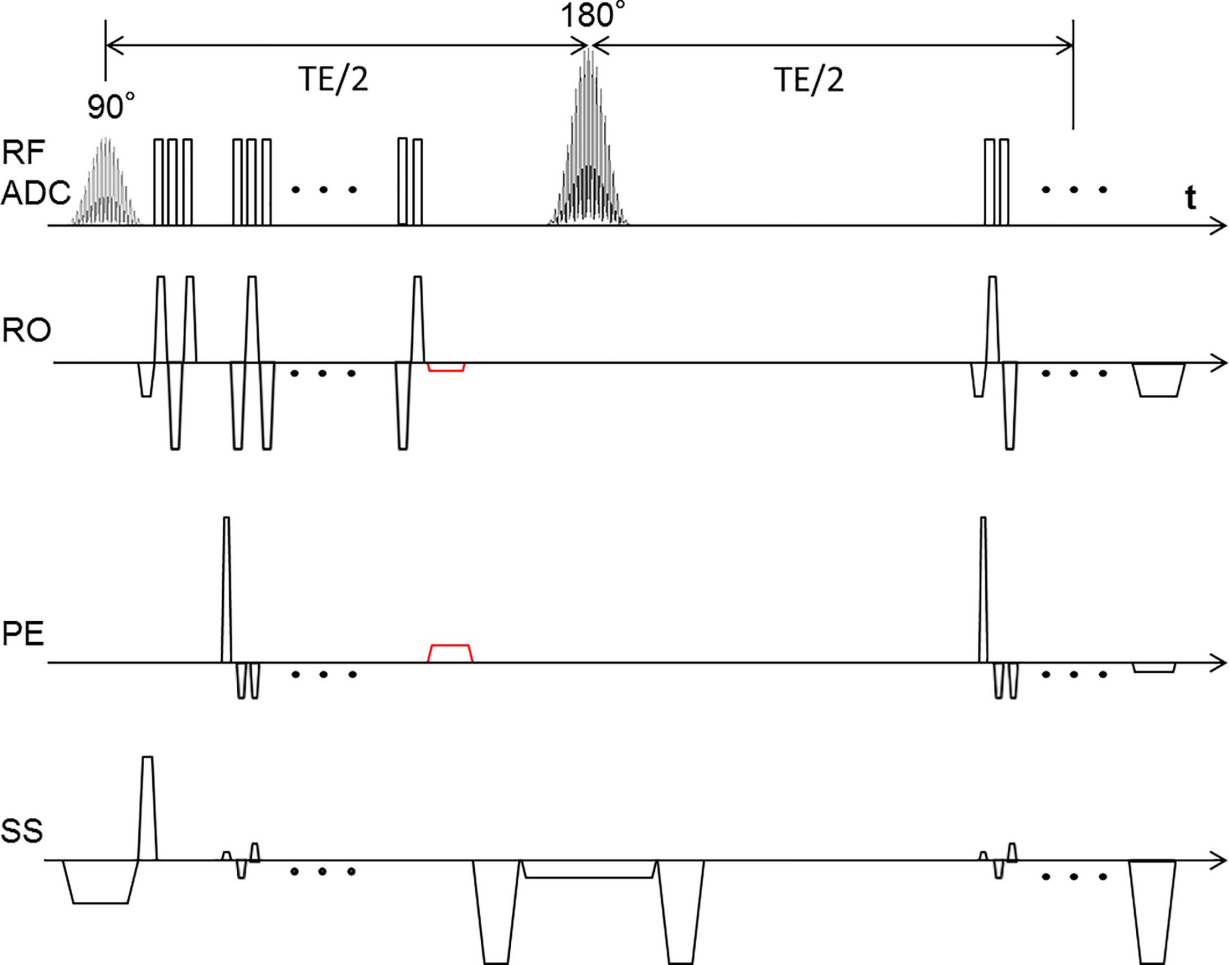
Sequence diagram of MEPI-SAGE. Several slices are exited simultaneously using MB RF. An additional gradient blip scheme in slice direction with each phase encoding step shifts the slices in the PE direction and reduces the high g-factor penalties and effectively reduces the noise amplification in the reconstructed images. The rephasing gradient (in red) was inserted after GE readout to return the data acquisition to the k-space center.

In this work, we use a multiband (MB) EPI accelerated VAI sequence to double the readout slices for whole-brain coverage. With the combination of blipped-controlled aliasing in parallel imaging (blipped-CAIPI), a low geometry (g)-factor penalty and high SNR can be achieved. The phases imprinted by the first EPI readout are thereby canceled by the rephasing gradients just before the refocusing pulse to easily enable in-plane parallel imaging (Fig 1).

## Methods

Data evaluation was approved by the local ethics committee of the University of Heidelberg (ethics approval number: S-320/2012), Heidelberg, Germany, and the requirement for informed consent was waived. All patients had consented to the scientific use of their data with admission to our hospital. They received scans using a 20-channel head receive RF coil on a 3T Prisma Siemens scanner (Siemens Healthcare, Erlangen, Germany), following a standardized tumor protocol at the department of neuroradiology at Heidelberg University Hospital, including FLAIR image acquisition, diffusion-weighted imaging, T2-weighted imaging, vessel architectural imaging during preload injection of intravenous gadoterate meglumine (Dotarem; Guerbet, France) and subsequent dynamic-susceptibility contrast imaging during contrast bolus injection, and pre- and postcontrast T1-weighted three-dimensional magnetization-prepared rapid acquisition gradient-echo imaging, see also [13, 14]. In total, 32 patients (11 women, 21 men; mean age ± standard deviation, 52.5±17.7) with low grade (14 patients) and high grade (18 patients) gliomas were included in this study, see Table 1. HGG patients with a time difference > 2.5 years between histopathologic diagnosis and MRI exam were excluded for the statistical analysis.

**Table 1:** Patient characteristics.

No.	Age	Sex	Glioma WHO	Location
1	37	M	II	left hemisphere
2	76	F	IV	right hemisphere
3	44	F	II	left hemisphere
4	51	M	IV	right hemisphere
5	52	M	II	left hemisphere
6	70	M	IV	multifocal, basal ganglia
7	67	M	IV	left hemisphere
8	78	M	IV	right hemisphere
9	49	M	IV	left hemisphere
10	36	M	II	left hemisphere
11	85	F	IV	left hemisphere
12	38	F	II	left hemisphere
13	58	F	II	left hemisphere
14	76	M	IV	right hemisphere
15	68	M	IV	left hemisphere
16	20	F	IV	left cerebello-pontine angle
17	43	F	IV	right hemisphere
18	34	M	II	right hemisphere
19	80	M	IV	left hemisphere
20	56	M	IV	right hemisphere
21	65	F	IV	left hemisphere
22	42	M	II	both hemispheres
23	76	F	IV	left hemisphere
24	57	M	IV	right hemisphere
25	62	M	IV	multifocal, basal ganglia
26	47	M	IV	right hemisphere
27	53	M	II	left hemisphere
28	37	F	II	left hemisphere
29	36	M	II	right hemisphere
30	24	F	II	right hemisphere
31	57	M	II	left hemisphere
32	25	M	II	right hemisphere

### Vessel architecture imaging

The MEPI-SAGE sequence is based on a clean EPI sequence; by inserting the rephasing gradient after GE EPI, a higher in-plane resolution can be easily achieved by exploiting a factor of 3 for the parallel imaging technique. Without the rephasing gradient, the actual position in k-space is flipped to the diagonal position of the k-space after application of a 180° pulse. However, by using parallel imaging acceleration, the start point and end point in k-space are not point-symmetric to the k-space center, so that the k-space position before the second EPI readout train, which depends on the acceleration factor, needs to be corrected. To ensure correct data acquisition, and to allow the usage of a standard EPI module, the phases produced by the EPI readout were canceled just before the refocusing pulse.

By using readouts of dual GE/SE 2D EPI, 60 measurements including 10 baseline measurements were obtained in a time of 1.5 minutes. Explicit sequence parameters were as follows: TE (GE/SE) = 22/90 ms, MB factor = 2, parallel imaging factor = 3, dim = 120×120, resolution = 2×2 mm^2^, TR = 1.5s. The slice thickness is 4.5 mm, distance factor is 20% (0.9mm) and slice number is 24. The total coverage of MB EPI is 13 cm. VAI examinations were performed with administration of 0.1 mmol/kg-bodyweight gadoterate meglumine (Dotarem, Guerbet) at a rate of 4 ml/s using an MR-compatible injector. A 20 ml bolus of saline was injected subsequently at the same rate. Singleband (SB) acquisition at the same slice position without injection of contrast agent was performed after MB acquisition. The slice number is 12 and the total coverage of SB EPI is 6.5 cm.

### Data processing

Before VAI analysis, motion correction including realignment and reslicing was achieved by using SPM (Wellcome Trust Center for Neuroimaging, UCL, London, UK). The relaxation rates ΔR(t)_2,SE_ and ΔR(t)_2,GE_ were calculated from the SE and GE data based on the following equation:

$${\Delta R(t)}_{2,XE}=-\frac{1}{{TE}_{XE}}∙\ln\frac{{S(t)}_{XE}}{S_{0,XE}}$$ (1)

where XE stands for SE or GE, respectively. TE is the echo time, S_0_ is the baseline signal from averaging of the first 10 signals before injection of preload contrast agent bolus, and S(t) represents the signal during the bolus passage of the VAI sequence. Only those voxels, whose peak signal, *p*, during preload contrast administration was larger than two times the sum of averaged baseline signal, μ, and baseline standard deviation, σ, or: *p* > 2[*μ* + σ], were included in further analysis. All other voxels were discarded. Contrast agent leakage effects were accounted for based on the method proposed by Boxerman et al. [15]. The corrected relaxation rate time curves were fitted in a gamma-variate function [16] and used for calculation of the ΔR(t)_2,SE_ versus ΔR(t)_2,GE_ diagram, producing a vascular hysteresis loop (VHL), see also [2]. The fitting procedure was based on the method of nonlinear least squares with a maximum iteration number of 4000; if fitting generated an error, the signal was set to zero. We excluded all fits with an r-value below 0.35. VAI quantification was performed using in-house developed MATLAB code (MathWorks, Natick, MA). Vessel size index and mean vessel density Q were calculated based on the procedure in [1, 2]. Since DTI was not included in our protocol, the ADC was set to the constant value of 0.8 μm^2^/ms [17].

To ensure readers of the robustness of the acquired data, one example of a voxel-wise uncorrected ΔR(t)_2,XE_ curves in enhancing voxels is presented in S1 Fig. GE and SE voxels that exhibited T1 leakage effects were identified as the voxels that exhibit signals that increase above the pre-contrast baseline and overlapped (S2 Fig). These results demonstrate that leakage effects needed to be corrected.

All VAI parameters can be calculated from the VHL: the maximum distance between ascending and descending branches of the loop (I); the long axis (slope length), short axis and signed area of the vortex curve (microvessel type indicator MTI), the gradient of the long axis (slope), as well as the vascular-induced bolus peak-time shift (VIPS), which represents the temporal shift between the time-to-peak of the SE- and GE-EPI signal curves.

### Statistics

For analysis of the VAI parameters, we determined the mean value and standard deviation at five different ROIs. The ROIs were manually drawn in HGG patients based on the FLAIR and the contrast-enhanced T1-weighted images, respectively, by two trained radiologists (FTK, CHZ). ROIs were placed in: (a) tumor, (b) peritumoral non-edematous tissue, (c) peritumoral edema, (d) contralateral normal appearing brain tissue (cNAB), and (e) necrosis. Examples of ROI placements are shown in S3 Fig. VAI parameters in tumor were compared with related values in peritumor, edema and cNAB using the Wilcoxon signed rank test (Table 2). The linear correlation coefficients (R) and P-values between two parameters in the tumor core (Table 3) and normal tissue (Table 4) were also determined. Bonferroni correction was performed for multiple comparisons.

**Table 2:** P-values for comparisons of VAI parameter distributions between HGG tumor core and peritumor, edema, and cNAB.

	Tumor↔Peritumor	Tumor↔Edema	Tumor↔cNAB
I	0.010	0.007	0.001
MTI	0.010	0.010	0.010
VIPS	0.041	0.121	0.008
Slope	0.188	0.421	0.330
Short Axis	0.015	0.041	0.188
Slope Length	0.022	0.064	0.421
rCBV	0.599	0.002	0.048

**Table 3:** Correlation coefficients and p-values between pairs of VAI parameter distributions in the tumor core (N = 15).

		**Correlation Coefficient**					
	I	MTI	VIPS	Slope	Short Axis	Slope Length	rCBV
I	1.000	0.864	0.768	-0.878	-0.642	-0.826	-0.545
MTI	0.864	1.000	0.639	-0.717	-0.780	-0.795	-0.469
VIPS	0.768	0.639	1.000	-0.570	-0.763	-0.485	-0.310
Slope	-0.878	-0.717	-0.570	1.000	0.563	0.919	0.730
Short Axis	-0.642	-0.780	-0.763	0.563	1.000	0.639	0.284
Slope Length	-0.826	-0.795	-0.485	0.919	0.639	1.000	0.633
rCBV	-0.545	-0.469	-0.310	0.730	0.284	0.633	1.000
		**P-value**					
	I	MTI	VIPS	Slope	Short Axis	Slope Length	rCBV
I	0.000	0.000	0.001	0.000	0.010	0.000	0.035
MTI	0.000	0.000	0.010	0.003	0.001	0.000	0.078
VIPS	0.001	0.010	0.000	0.027	0.001	0.067	0.261
Slope	0.000	0.003	0.027	0.000	0.029	0.000	0.002
Short Axis	0.010	0.001	0.001	0.029	0.000	0.010	0.305
Slope Length	0.000	0.000	0.067	0.000	0.010	0.000	0.011
rCBV	0.035	0.078	0.261	0.002	0.305	0.011	0.000

**Table 4:** Correlation coefficients and p-values between pairs of VAI parameter distributions in the normal tissue (N = 15).

		**Correlation Coefficient**					
	I	MTI	VIPS	Slope	Short Axis	Slope Length	rCBV
I	1.000	0.838	0.753	-0.078	0.078	0.093	0.335
MTI	0.838	1.000	0.455	0.148	-0.182	0.194	0.491
VIPS	0.753	0.455	1.000	-0.200	0.281	-0.029	0.047
Slope	-0.078	0.148	-0.200	1.000	-0.428	0.660	0.668
Short Axis	0.078	-0.182	0.281	-0.428	1.000	0.083	-0.190
Slope Length	0.093	0.194	-0.029	0.660	0.083	1.000	0.642
rCBV	0.335	0.491	0.047	0.668	-0.190	0.642	1.000
	**P-value**						
	I	MTI	VIPS	Slope	Short Axis	Slope Length	rCBV
I	0.000	0.000	0.001	0.781	0.782	0.741	0.222
MTI	0.000	0.000	0.089	0.597	0.517	0.488	0.063
VIPS	0.001	0.089	0.000	0.476	0.311	0.918	0.869
Slope	0.781	0.597	0.476	0.000	0.111	0.007	0.006
Short Axis	0.782	0.517	0.311	0.111	0.000	0.770	0.499
Slope Length	0.741	0.488	0.918	0.007	0.770	0.000	0.010
rCBV	0.222	0.063	0.869	0.006	0.499	0.010	0.000

## Results

### Multiband and singleband EPI produce similar images

**Fig 2:**
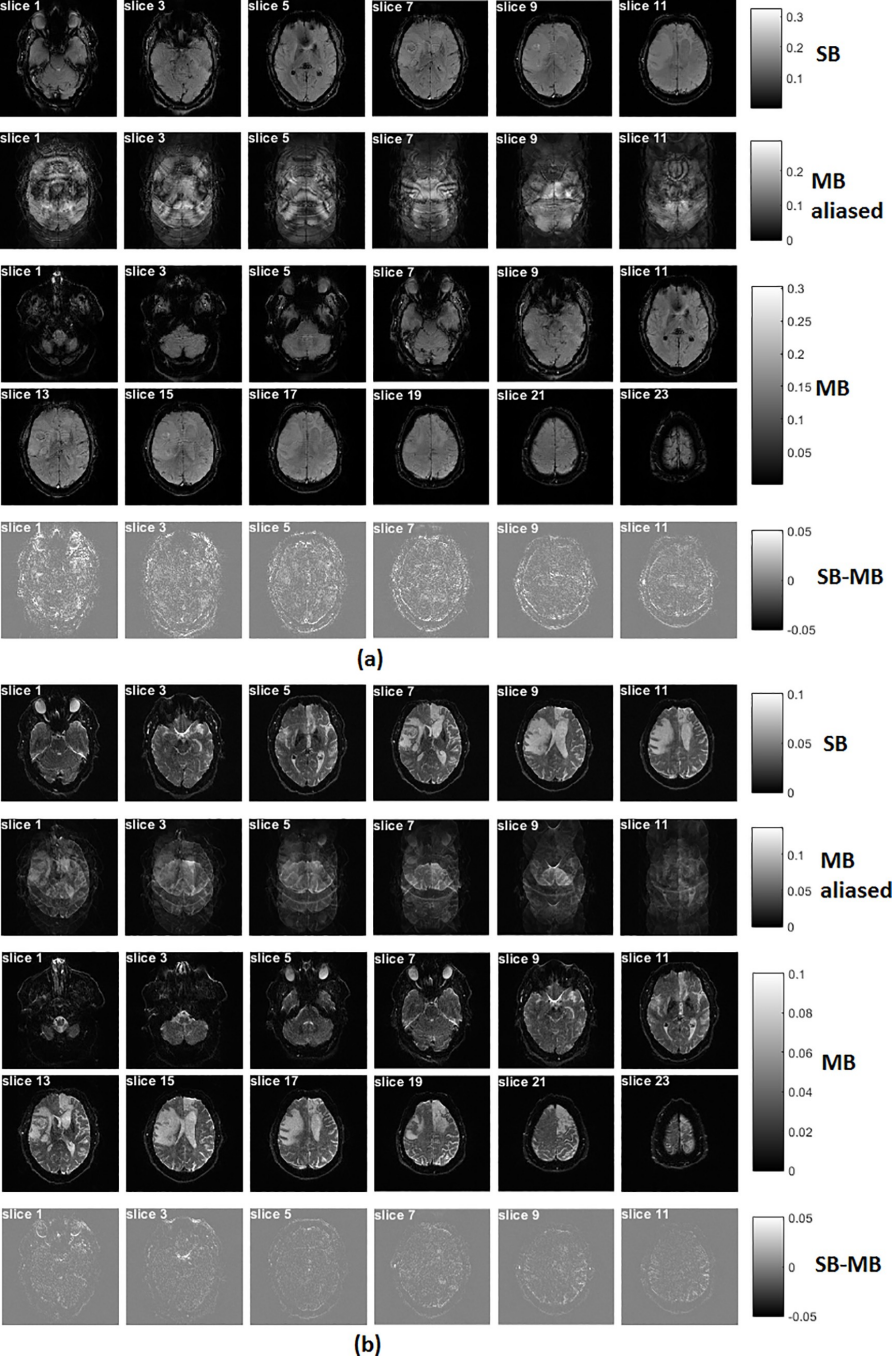
GE (a) /SE (b) EPI-based images acquired with single and multiband excitations. All images were reconstructed with an unfolding procedure for three-fold in-plane parallel imaging. With use of the multiband technique, the number of slices was increased to 24 (versus 12 slices in the singleband data). Using a multiband excitation with a multiband factor of 2 as well as blipped-CAIPI, two simultaneously excited slices were folded together with a phase shift. After reconstruction with the 'Slice-GRAPPA' algorithm [19], these two-folded slices can be clearly separated. Subtraction of multiband EPI from singleband EPI produces only little differences at the same slice location.

When compared to the SB technique, the MB produces multiple clones of the original SB profile, each of which has a symmetric frequency offset [18]. Fig 2 shows the EPI-based images acquired with the SB and MB techniques, respectively. A representative reconstructed volume is presented for each data set. All images were reconstructed after the unfolding procedure was applied to account for the 3-fold in-plane acceleration, which was introduced using parallel imaging. Two simultaneously excited slices are aliased to be contained in one slice with the use of the MB excitation. After the ‘Slice-GRAPPA’ reconstruction [19], the two simultaneously acquired slices were successfully separated from the MB-aliased images. Subtraction of MB EPI from SB EPI produces little differences at the same slice location (Fig 2). Presumably, this difference is mainly induced by motion during imaging.

### Signal-to-noise ratios slightly lower in multiband imaging

The SNR values were calculated by 0.65×S/ σ, with S being the mean pixel intensity value in brain, and σ being the noise-associated standard deviation of background air, employing a correction factor of 0.65 for background noise (Rician distribution) [20]. The SNR of the reconstructed images from all subjects at the same slice position are listed in S1 Table. The mean SNR are 106.6 and 101.3 for SB and MB in GE EPI, and 38.2 and 36.8 for SB and MB in SE EPI, respectively.

### G-factor maps and image histograms

**Fig 3:**
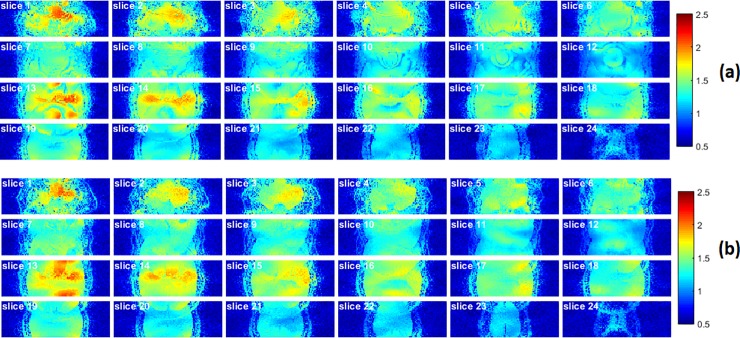
G-factor maps of GE (a) and SE (b), induced from the MB acceleration. The g-factor noise penalty, induced from the MB acceleration of all subjects, has an averaged value of 1.35 and 1.33 for GE and SE imaging, respectively. This relative low g-factor was achieved using a blipped-CAIPI technique.

The g-factor maps of GE and SE were calculated after the application of MB data reconstruction (Fig 3). The g-factor values induced from the MB acceleration of all subjects has an averaged value of 1.35 for GE and 1.33 for SE (S2 Table).

**Fig 4:**
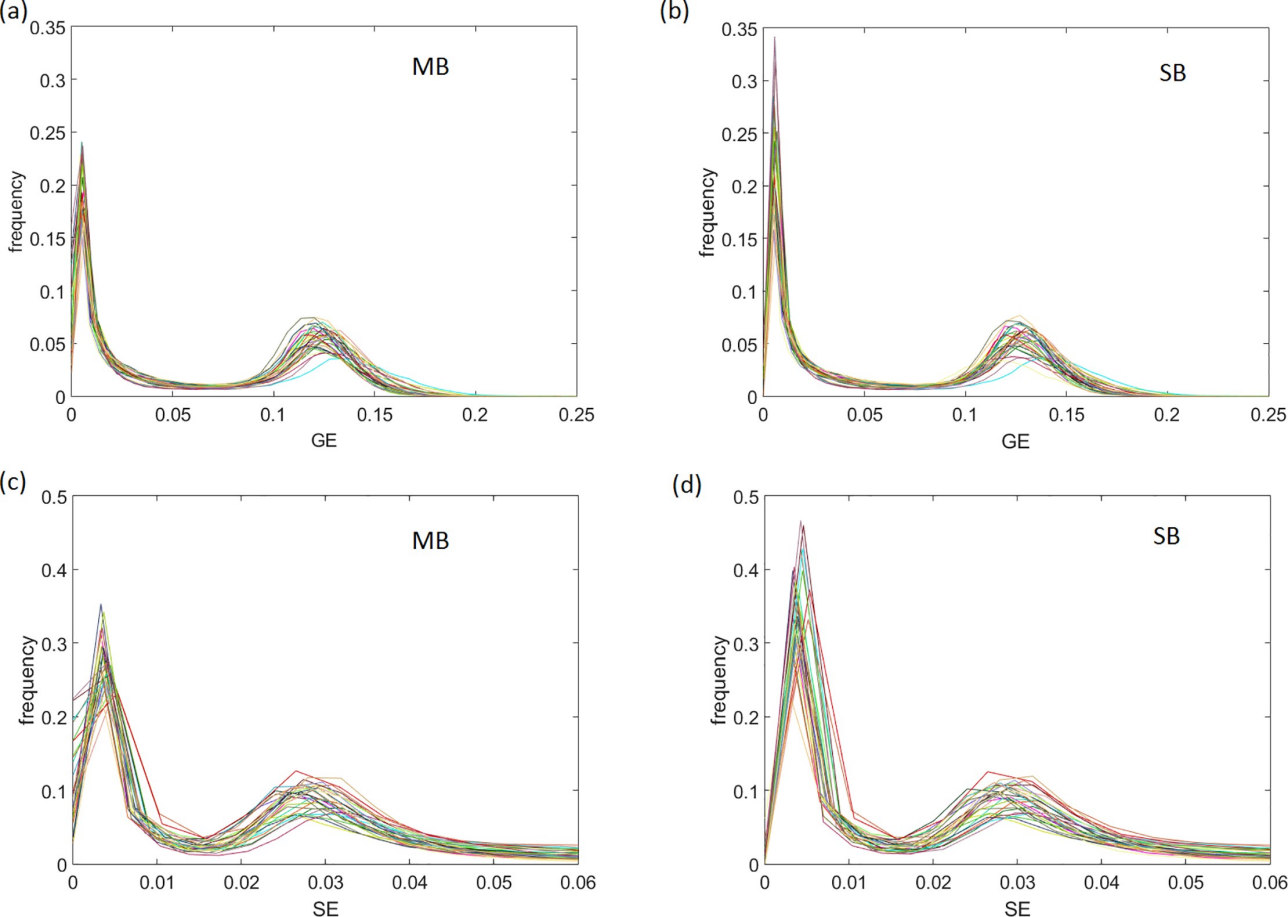
Histograms of equally located image slabs for GE readout intensity using multiband (MB) (a) and singleband (SB) (b) techniques, and for SE readout intensity using MB (c) and SB (d) techniques. The averaged correlation coefficient for MB and SB histograms across all patients was found as 0.96 ± 0.03 for GE, and 0.96 ± 0.04 for SE readout intensity, respectively (N = 32).

The histograms of the image slabs at the same location from 32 subjects show similar distributions between MB and SB techniques in GE and SE readout intensity (Fig 4). We determined the correlation coefficient between MB and SB histograms for each patient for both GE and SE readout to find an averaged correlation coefficient of 0.96 ± 0.03 for GE readout intensity and 0.96 ± 0.04 for SE.

### Vascular hysteresis loops

**Fig 5:**
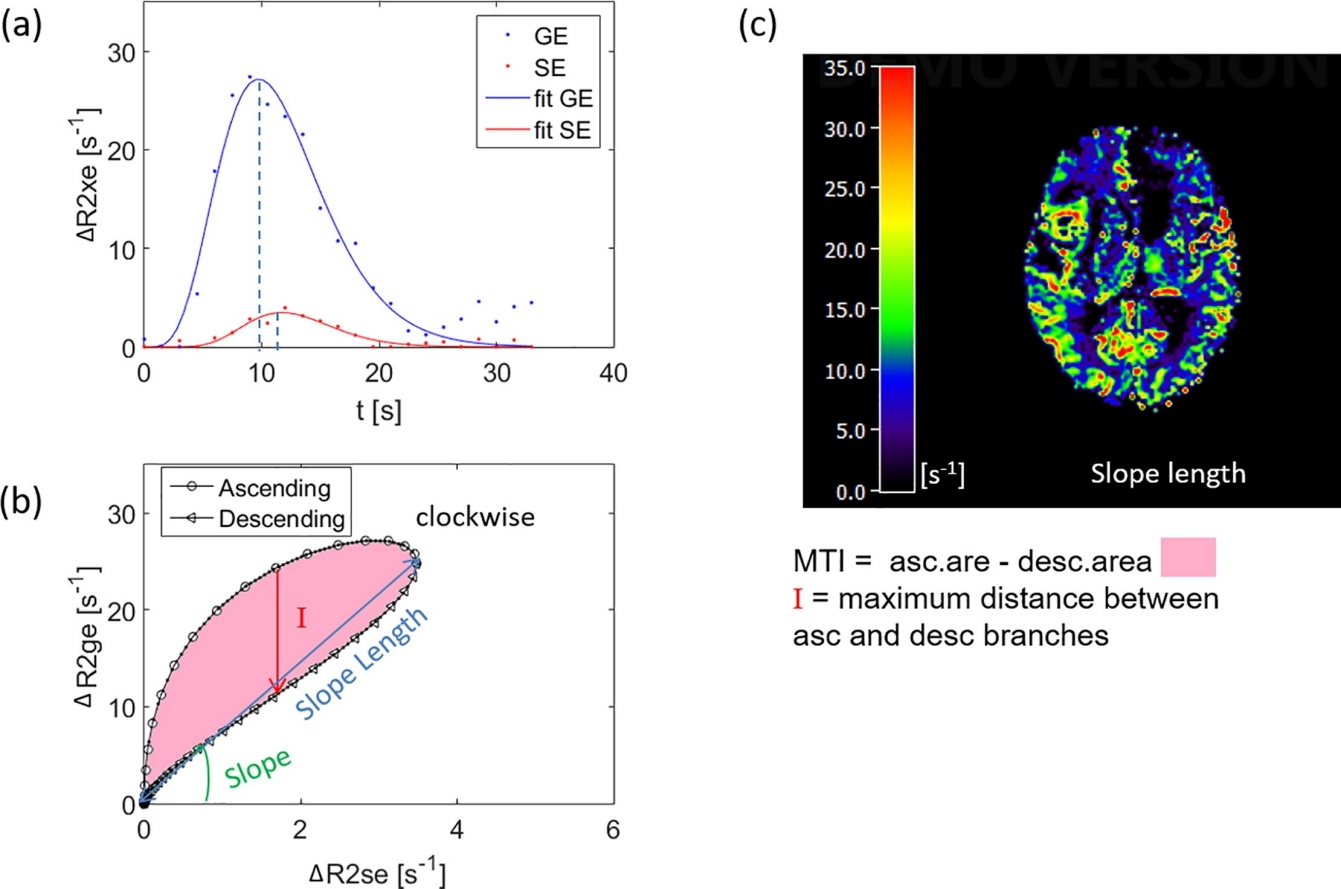
(a) Relaxation rate time curves from the voxel in the region of the tumor core. The GE signal peaks earlier than the SE signal, resulting in a clockwise loop when plotting the relaxation rate curves in a point-by-point time-parametrized plot (b). (c) The slope length map visualizes the long axis of the loop. (XE = GE, SE; see also Eq (1) in the methods section).

A VHL from a voxel at the region of the tumor core is represented in Fig 5 for a patient with glioblastoma multiforme. To illustrate the variability of VHLs in tumor regions, we show VHL curves in three different tumor areas for the patient with glioblastoma (WHO IV; S4 Fig), another patient with glioblastoma (WHO IV; S5 Fig) and a patient with oligodendroglioma (WHO II; S6 Fig). After plotting the relaxation rate curves in a point-by-point time-parametrized plot, we found a clockwise loop that is typical for large arterial inflow, as shown previously [1, 2].

### VAI parametric maps

**Fig 6:**
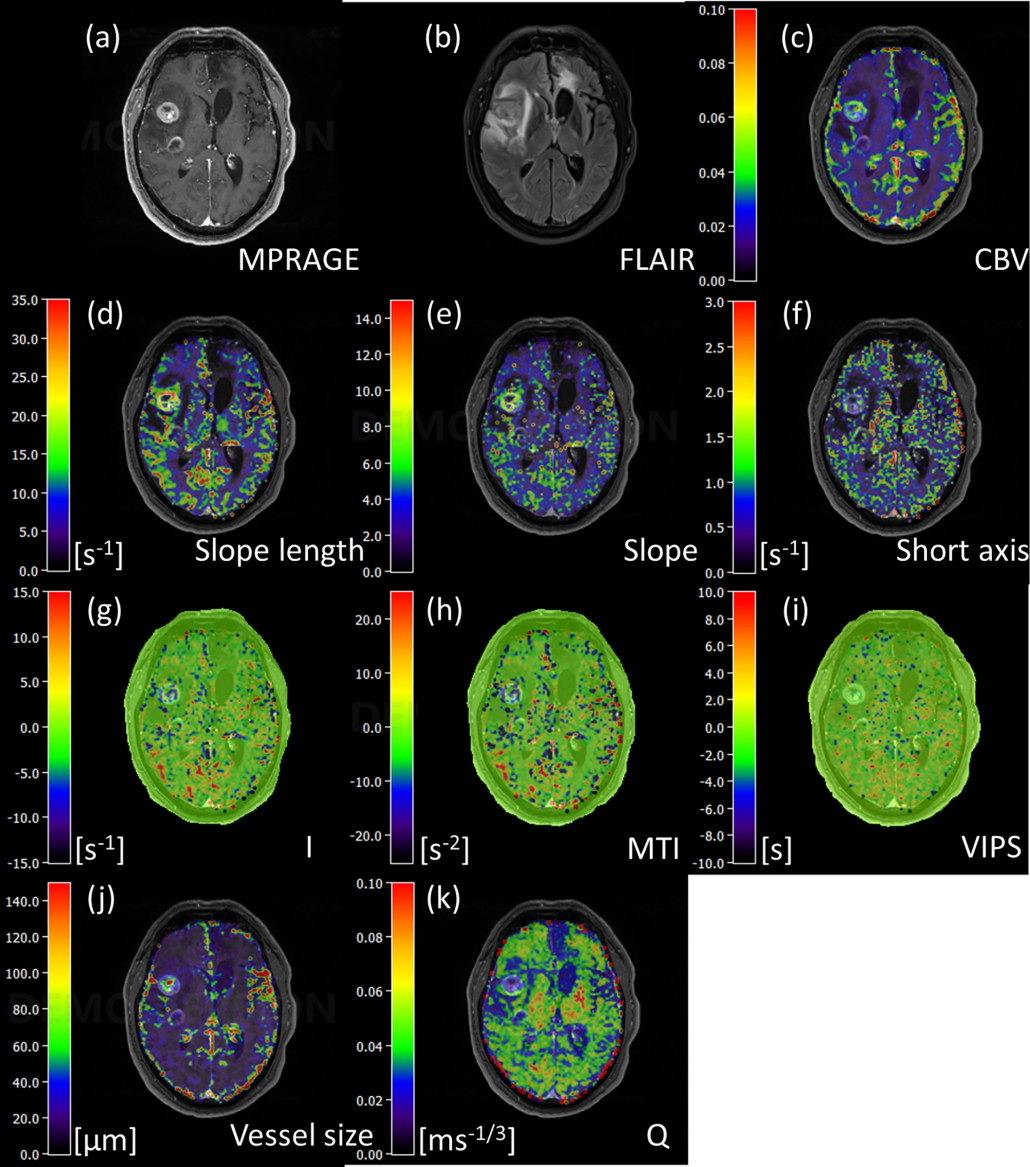
(a) Contrast-enhanced T1-weighted MPRAGE image. (b) FLAIR image. (c) rCBV. (d) Slope length. (e) Short axis. (f) Slope. (g) Distance map. (h) MTI. (i) VIPS. (j) Vessel size. (k) Q map.

Maps of VAI parameters from the same patient are represented in Fig 6. More cases of parametric maps can be found in S7 and S8 Figs. For instance, in S7 Fig, there is a strong decrease in distance map and MTI parameters in the tumor core area. The maps for slope length, slope and short axis are parameter maps based on absolute values, whereas parameter maps for I, MTI and VIPS are signed to account for the direction of the VHL. The VHL in a voxel transverses in the clockwise direction if the vascular system in this voxel contains mostly arterioles and capillaries, i.e. in case of relatively higher arterial blood volume content, whereas the VHL transverses in a counterclockwise direction if the vascular system in this voxel consists of mainly venule- and capillary-like vessel components, corresponding to a relatively higher venous blood volume content, see also [2, 3, 21]. According to the definition for the calculation of I and MTI, a clockwise VHL-direction was associated with a positive signed distance and signed area, i.e. positive I and MTI, and vice-versa for the counterclockwise VHL direction. In the maps of I and MTI, negative values were assigned to cool colors and positive to warm colors, respectively. A voxel with high arterial blood volume shows positive I and MTI values and an orange to red color in I and MTI maps; a capillary voxel shows low I and MTI values and green color in the maps; and a voxel with high venous blood volume shows negative I and MTI values and blue to purple color in the maps. In tissue areas that predominantly host venules and capillaries, the SE-EPI perfusion signal peaks earlier than the GE-EPI perfusion signal, corresponding to a negative VIPS value, and vice-versa for arteriole-dominated microvasculature. In fact, one can see that the distance maps display many dots of extreme colors, that typically show large arteries and veins, see also S9 Fig, where we demonstrate this effect, based on an additional SWI and TOF image of the same axial slice location as the distance map (see legend to S9 Fig for details).

In the VIPS maps, similarly to I and MTI, negative VIPS values were assigned to cool colors and positive to warm colors, respectively. In Fig 6G and 6H, we see an increase in negative I and MTI values in the larger contrast-enhancing tumor in the right frontal lobe, indicating a relatively venous/venule blood volume content than in the surrounding tissue. However, the smaller ring-enhancing tumor component with central necrosis, that is located posterior to the frontal bulk tumor, shows a more mixed microvascular architecture with partly venous and partly arterial-dominated blood volume content voxels, indicating a different state of angiogenesis. This distinction could not have been made based on the rCBV map alone that only shows an increased blood volume content in both bulk tumors.

In fact, among the parametric maps, the slope length map features the most similar contrast to rCBV: both maps exhibit increased value areas in the bulk tumor and decreased value areas in the surrounding edematous region, see Fig 6C and 6D. This is highly suggestive of a strong correlation (p = 0.011) between VHL slope length and rCBV, as confirmed for the bulk tumor region in Table 3.

Vessel size index (VSI) and parameter Q, a measure of microvessel density, are shown in Fig 6J and 6K for the same patient. The VSI is understood as a mean vessel radius averaged over the capillary population with the weight of its volumetric fraction; therefore, it can be used to monitor the dilation and contraction of microvessels [22]. The increase of VSI in the bulk tumor (Fig 6j) corresponds to a decrease of Q (Fig 6k). Values of VSI in the tumor core range between 90–140 μm, similar to results in previous study [23].

### Statistical results

**Fig 7:**
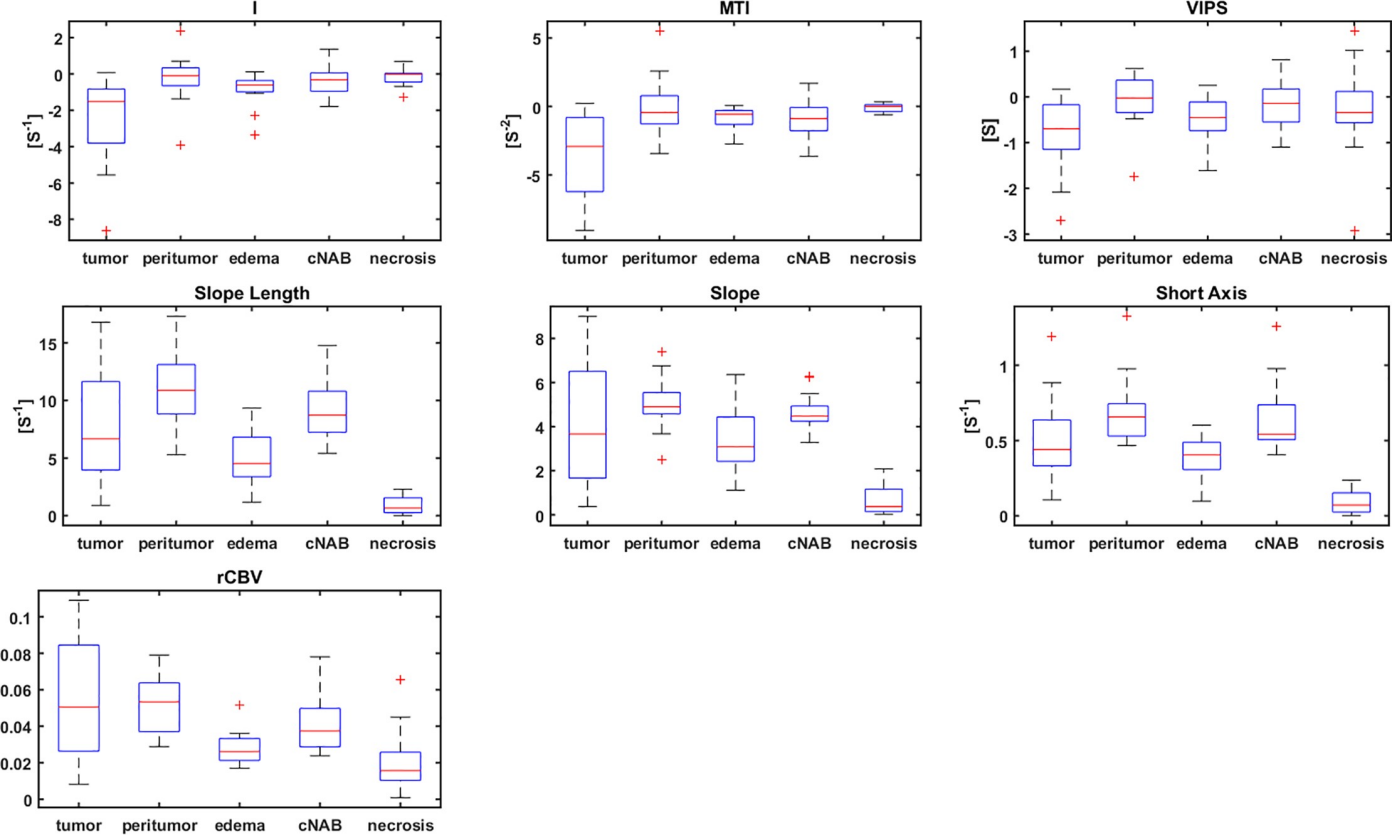
Box-whisker plots (whiskers: Minimum, maximum; box: 25th to75th percentile; line: Mean value) for VAI parameters I, MTI, VIPS, slope length, slope; short axis, rCBV in tumor core; peritumor (non-edematous); edema; cNAB.

**Fig 8:**
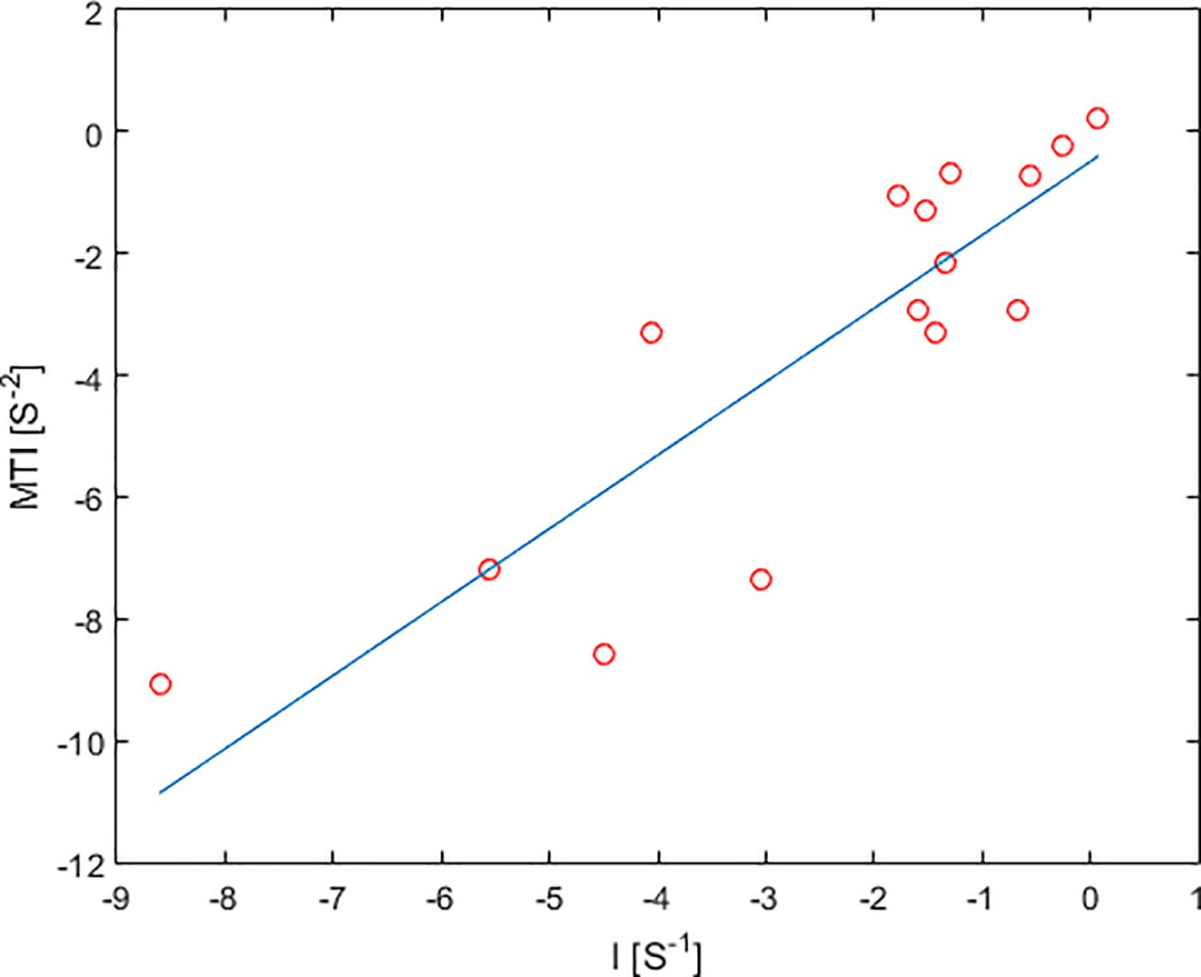
Linear correlation between VAI parameters I and MTI in the tumor core of HGG (red circle).

Within the HGG patient collective (N = 15), VAI parameters I and MTI were lower in the tumor core than in the peritumor, edema and cNAB regions (Fig 7, Table 2). VIPS in the tumor core for HGG patients was significantly decreased to the peritumor (p = 0.041) and cNAB regions (p = 0.008). Similarly, the short axis in the HGG tumor core was significantly decreased to the peritumor region (p = 0.015) and increased to edema (p = 0.041), the slope length in the tumor core was significantly increased to edema (p = 0.022), and, furthermore, tumor core rCBV was significantly increased to edema (p = 0.002) and cNAB (p = 0.048). A positive linear correlation between VAI parameters I and MTI were found both in the tumor core of HGG (Fig 8). Furthermore, parameter distributions of I, MTI and VIPS in the bulk tumor showed were highly correlated, see Table 3 (p<0.01). In fact, one can clearly see that the maps of I, MTI and VIPS are similar, see Fig 6, S7 and S8 Figs. Likewise, slope, short axis and slope length in the bulk tumor were correlated (p<0.03; see Table 3) and parametric maps of slope, short axis and slope length looked similar, see Fig 6, S7 and S8 Figs. In addition, CBV was found as the parameter to have no correlation with VAI parameters I, MIT, VIPS and short axis (p>0.08), but showed a high correlation with slope length. This can be explained by the slope length representing the root sum square of the peaks of the two relaxation rate curves, while rCBV is proportional to the area under the GE relaxation rate curve, which is determined by the peak of the GE relaxation rate curve. Similar to the tumor core, VAI parameters I, MTI and VIPS in normal tissue are in part correlated; however, MTI and VIPS are not correlated (p = 0.09). In addition, in normal tissue, only slope and slope length are highly correlated (p<0.01), whereas short axis is not.

## Discussion

In this work, the MB technique was integrated in a simultaneous GE/SE EPI sequence for VAI imaging to increase the number of slices in order to achieve whole-brain coverage. The optimized GE/SE EPI sequence achieves twice the number of slices whilst maintaining the same repetition time (1.5s) as the SB technique. In previous studies with simultaneous GE/SE EPI based VAI, only a few slices could be acquired for the dynamic tracking of relaxation rates [1, 2, 24]. Generally, in order to capture the signal changes after the injection of contrast agent, there is a trade-off between temporal resolution and number of slices such that the temporal resolution needs to be reduced if more slices per readout are to be acquired. The use of simultaneous multi-slice imaging not only provides better tissue coverage, it also allows for modifications of the TR. In theory, a shorter TR should allow for a better sampling of the VAI vortex curve.

To achieve high SNR, high spatial resolution, and coverage of the whole brain, previous works utilized a dual contrast agent injection approach in combination with two separate SE and GE acquisitions to determine VAI parameters [4, 21, 23]. However, patient motion between two scans as well as differences in the time to peak for the two bolus injections may significantly affect data evaluation. Additionally, the dual contrast agent injection approach effectively doubles the dose of contrast agent together with a significant increase in measurement time, when compared to simultaneous GE/SE EPI acquisitions. Furthermore, leakage effects of contrast agent need to be corrected.

Applications of the MB technique have also been demonstrated previously for dynamic susceptibility contrast (DSC) imaging [12], where the authors applied MB excitation and blipped-CAIPI to a GE/SE EPI sequence with the same MB factor but lower in-plane iPAT factor of 2. However, by inserting the rephasing gradient after GE EPI, a higher in-plane resolution can easily be achieved by exploiting a higher iPAT factor of 3 for parallel imaging, as used in our study. Further optimization of MB RF to reduce peak power needs to be developed [25–27]. In this study, we employed a MB factor of 2 due to the relative low number of channels of our RF coil (20). A higher MB factor would reduce the TR for the same brain coverage and would also help to make the resolution more isotropic. Generally, the MB technique has been widely applied in the field of MR perfusion. MB with multi-echo EPI (3 and 7 echoes, respectively) has been applied to assess perfusion and permeability [28] and in hyperpolarized ^13^C spectroscopic imaging [29]. MB acquisition was also successfully applied in ASL [30].

As shown above, the quality of the images resulting from MB acceleration is comparable to those from the SB method, see Fig 2. The images were reconstructed without any loss of spatial resolution or severe distortions, and, in addition, MB and SB SNR were similar.

We have acquired VAI parameter maps from a patient collective with HGG tumors. In agreement with results from a previous study [4], we found that MTI in the tumor core of HGG was decreased when compared to peritumor, edema and cNAB (Table 2). Since MTI values are sensitive to the temporal shift between SE- and GE-perfusion, induced by changes in the vascular architecture, as well as the blood volume [2, 3], the observed decreased values in hyperperfused tumor tissue is likely dominated by the strong increase in blood volume. In contrast, VIPS in the tumor core of HGG was decreased when compared to peritumor and CNAB, but not to edema. Presumably, VIPS is less sensitive to blood volume, when compared to MTI, but more sensitive to early neovascularization [4], and, thus, provides additional information at the tumor periphery. A decreased VIPS in peritumoral edema may therefore indicate neovascular changes, e.g. vascular co-option through advancing tumor cells.

In addition, we found a strong correlation between VAI parameters I, MTI and VIPS in the tumor core, while MTI and VIPS in normal tissue were not significantly correlated. This again indicates that the strong increase in blood volume in the tumor core due to hyperperfused tumor tissue may have a more profound effect on MTI and VIPS values than changes in vascular architecture. Similarly, we also found a strong correlation in VAI parameters slope and slope length in both tumor core and normal tissue, as opposed to VAI parameter short axis, that does not correlate in normal tissue with either slope or slope length. This again indicates that the specific circumstances of increased blood volume and changes in vascular architecture of the tumor core are dominated by blood volume effects, thus allowing for a reduction in VAI parameters to characterize this phenomenon, e.g. MTI and slope length, while an extensive discrimination of vascular changes in healthy tissue and tumor periphery needs a more careful consideration of additional VAI parameters such as VIPS and short axis.

The increased temporal resolution as well as the improved brain coverage offered by a multiband dual echo GE/SE can also be a great asset for functional studies of the healthy brain. In fact, recent studies could show that GE and SE capture different features of the BOLD response respectively (e.g. a different linearity of the response or an increased benefit of SE for magnetic field inhomogeneities), see [31–33].

In conclusion, the high-resolution GE/SE EPI vessel architectural imaging sequence provides significantly improved slice coverage, and the corresponding VAI parametric maps offer more detailed insights into the complexity and heterogeneity of vascular changes in glioma.

## Supporting information

S1 Table SNR comparison. The SNR of singleband and multiband for GE and SE readouts, respectively, were calculated for all subjects.
(PDF)

S2 Table G-factor comparison. The averaged g-factors of GE and SE readouts induced from the multiband technique were calculated for all subjects.
(PDF)

S1 Fig Example of uncorrected ΔR(t)_2,XE_ curves in an contrast-enhancing voxel of a right-hemispheric tumor (blue cross). (TIF)

S2 Fig GE and SE voxels, that exhibited T1 leakage effects, are shown in colors gray (GE) and red (SE), respectively. Overlapping voxels are shown in green. (TIF)

S3 Fig Definition of region of interests (ROI). Respective ROIs for three patients are shown on contrast-enhanced T1-weighted MPRAGE images (left hand side) and FLAIR images (right hand side), where the patient in (a) has a glioblastoma (WHO IV) and patient (b) a glioblastoma (WHO IV). Tumor ROIs (in red) were placed at around the contrast-enhancing lesions on the T1-weighted MPRAGE images. Peritumoral edema ROIs (in pink) were placed in representative FLAIR-hyperintense areas neighboring the contrast-enhancing tumor lesions. Peritumoral non-edematous tissue ROIs (in blue) were placed in close vicinity to the contrast-enhancing lesions in a brain area that did show neither contrast-enhancing lesions nor FLAIR-hyperintense lesions. The cNAB ROIs (in yellow) correspond to the mirrored tumor ROI on the contralateral side.
(TIF)

S4 Fig VHLs in a patient with glioblastoma (WHO IV). The left column shows relaxation rate time curves taken from three different voxels that are marked with crosses (from top to bottom: red, blue, cyan) in the T1-weighted MPRAGE image with overlaid FLAIR image on the right-hand side. When visualized in a time-parameterized plot, the pairwise GE and SE data points form a vortex curve of a certain shape and in either a clockwise or a counterclockwise direction, see the respective diagrams in the middle column.
(TIF)

S5 Fig VHLs in a patient with glioblastoma (WHO IV). The left column shows relaxation rate time curves taken from three different voxels that are marked with crosses (from top to bottom: red, blue, cyan) in the T1-weighted MPRAGE image with overlaid FLAIR image on the right-hand side. When visualized in a time-parameterized plot, the pairwise GE and SE data points form a vortex curve of a certain shape and in either a clockwise or a counterclockwise direction, see the respective diagrams in the middle column.
(TIF)

S6 Fig VHL in a patient with oligodendroglioma (WHO II). The left column shows relaxation rate time curves taken from three different voxels that are marked with crosses (from top to bottom: red, blue, cyan) in the T1-weighted MPRAGE image with overlaid FLAIR image on the right-hand side. When visualized in a time-parameterized plot, the pairwise GE and SE data points form a vortex curve of a certain shape and in either a clockwise or a counterclockwise direction, see the respective diagrams in the middle column.
(TIF)

S7 Fig VAI parametric maps in a patient with glioblastoma (WHO IV). (a) Contrast-enhanced T1-weighted MPRAGE image. (b) FLAIR image. (c) rCBV. (d) VAI slope length. (e) VAI short axis length. (f) VAI slope. (g) VAI distance map. (h) VAI MTI. (i) VAI VIPS. (j) VAI vessel size. (k) VAI Q. For further details, please see main text.
(TIF)

S8 Fig VAI parametric maps in a patient with oligodendroglioma (WHO II). (a) Contrast-enhanced T1-weighted MPRAGE image. (b) FLAIR image. (c) rCBV. (d) VAI slope length. (e) VAI short axis length. (f) VAI slope. (g) VAI distance map. (h) VAI MTI. (i) VAI VIPS. (j) VAI vessel size. (k) VAI Q. For further details, please see main text.
(TIF)

S9 Fig Extreme values in the VAI distance map. Extreme colors in VAI maps are usually linked to large arteries and veins: intraparenchymal large veins are easily identified on the SWI image (marked with a black arrow; left hand side image) and correspond to large negative values on the distance map (I) (middle image) close to -15s^-1^. Extreme positive values on the distance map, however, typically correspond to large arteries, e.g. branches of the posterior cerebral artery that were identified on the TOF image (right-hand side of the image) of the same axial slice location (white arrows): corresponding values in the distance map are close to +15s^-1^.
(TIF)
