# Supplementary material for: Vessel architecture imaging using multiband gradient-echo/spin-echo EPI
Source: PLoS One. 2019 Aug 9;14(8):e0220939. doi: 10.1371/journal.pone.0220939 (PMC6688807; doi:10.1371/journal.pone.0220939)
Supplement: S1 Table — The SNR of singleband and multiband for GE and SE readouts, respectively, were calculated for all subjects. (PDF) [file pone.0220939.s001.pdf]

No.	Singleband GE	Muliband GE	Singleband SE	Multiband SE
1	93,62	88,65	33,07	31,88
2	118,34	113,93	42,57	41,51
3	134	128,72	44,22	42,63
4	103,85	97,31	33,98	32,18
5	101,83	95,23	33,95	32,74
6	98,39	91,78	34,3	32,02
7	92,35	88,22	40,82	39,29
8	101,08	95,78	38,48	37,01
9	117,17	111,45	37,97	36,75
10	75,6	71,57	33,97	33,41
11	129,89	123,95	42,25	40,61
12	137,08	130,15	47,7	45,07
13	114,6	108,39	43,19	41,54
14	80,84	76,05	36,86	35,08
15	82,11	76,27	39,15	37,16
16	117,91	111,41	38,85	36,81
17	104,29	106,69	35,57	36,74
18	95,51	88,84	33,89	32,58
19	100,08	95,06	37,17	36,02
20	95,44	89,77	35,03	33,29
21	131,36	125,8	45,84	43,89
22	120,53	111,58	47,1	45,01
23	106,1	106,33	47,32	48,51
24	93,62	90,8	33,07	32,49
25	113,09	105,75	38,8	36,85
26	84,47	82,18	32,97	30,79
27	107,93	101,43	31,77	29,91
28	128,83	121,32	39,26	36,8
29	105,45	99,74	38,52	36,75
30	106	100,22	35,3	34,15
31	107,4	102,36	33,76	32,11
32	112,2	105,77	36,14	34,94
Mean	106,59	101,33	38,21	36,77
Std.	15,53	14,97	4,61	4,63
