# Supplementary material for: Vessel architecture imaging using multiband gradient-echo/spin-echo EPI
Source: PLoS One. 2019 Aug 9;14(8):e0220939. doi: 10.1371/journal.pone.0220939 (PMC6688807; doi:10.1371/journal.pone.0220939)
Supplement: S2 Table — The averaged g-factors of GE and SE readouts induced from the multiband technique were calculated for all subjects. (PDF) [file pone.0220939.s002.pdf]

G-factor Comparison

No.	GE	SE
1	1,3	1,26
2	1,36	1,36
3	1,64	1,64
4	1,21	1,2
5	1,27	1,25
6	1,32	1,28
7	1,42	1,4
8	1,4	1,39
9	1,41	1,39
10	1,22	1,2
11	1,52	1,49
12	1,28	1,28
13	1,25	1,23
14	1,31	1,3
15	1,31	1,28
16	1,2	1,18
17	1,37	1,34
18	1,2	1,16
19	1,44	1,4
20	1,34	1,33
21	1,3	1,27
22	1,34	1,29
23	1,54	1,51
24	1,25	1,21
25	1,39	1,36
26	1,53	1,49
27	1,32	1,33
28	1,41	1,41
29	1,29	1,3
30	1,49	1,47
31	1,25	1,24
32	1,37	1,36
Mean	1,35	1,33
Std.	0,11	0,11
