# Supplementary figures and images for: Vessel architecture imaging using multiband gradient-echo/spin-echo EPI
Source: PLoS One. 2019 Aug 9;14(8):e0220939. doi: 10.1371/journal.pone.0220939 (PMC6688807; doi:10.1371/journal.pone.0220939)

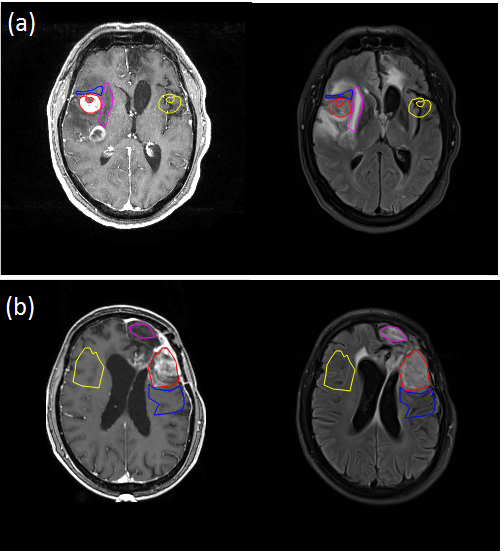

Supplement: S1 Fig — (TIF) [file pone.0220939.s003.tif]

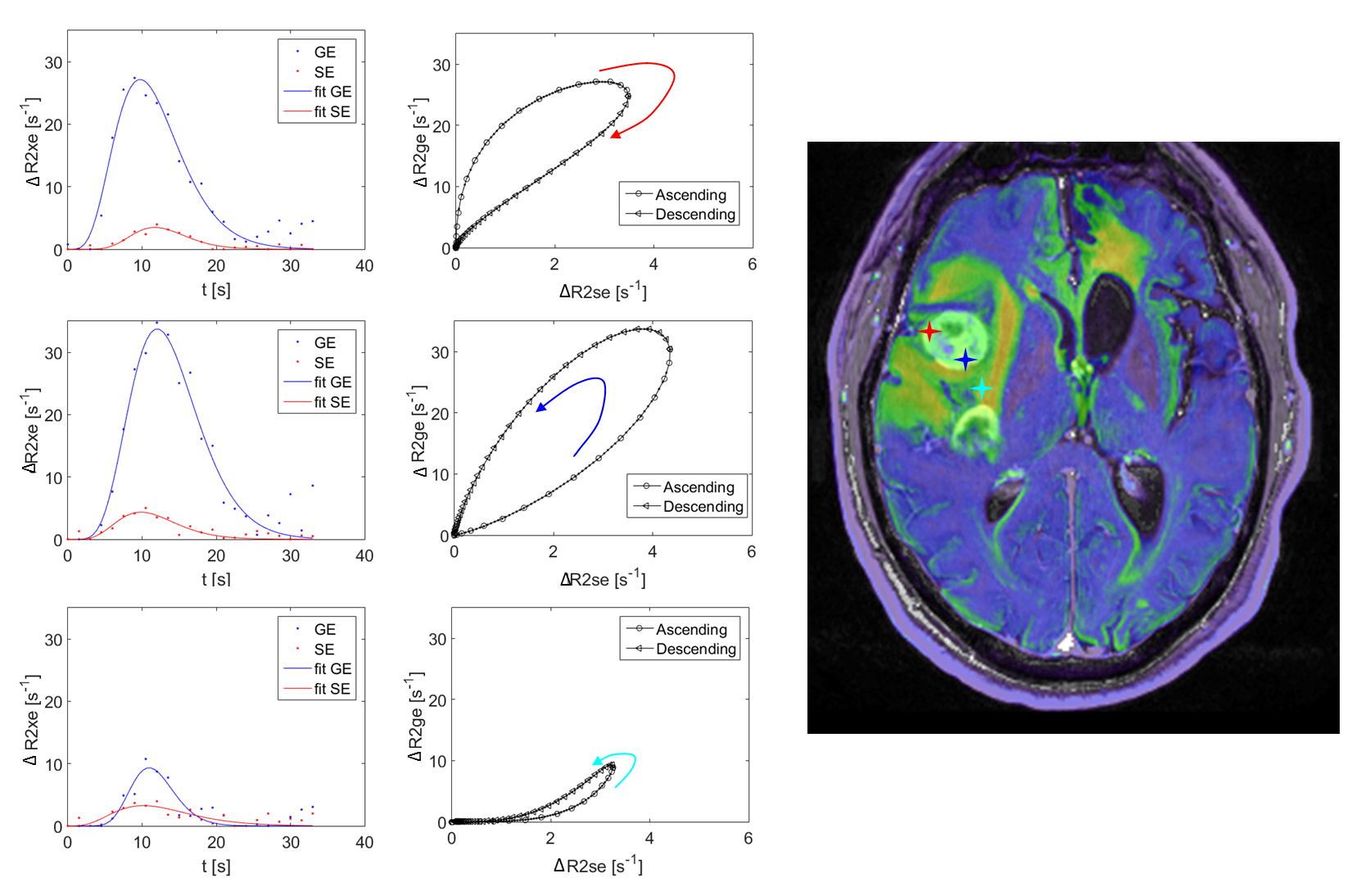

Supplement: S2 Fig — (TIF) [file pone.0220939.s004.tif]

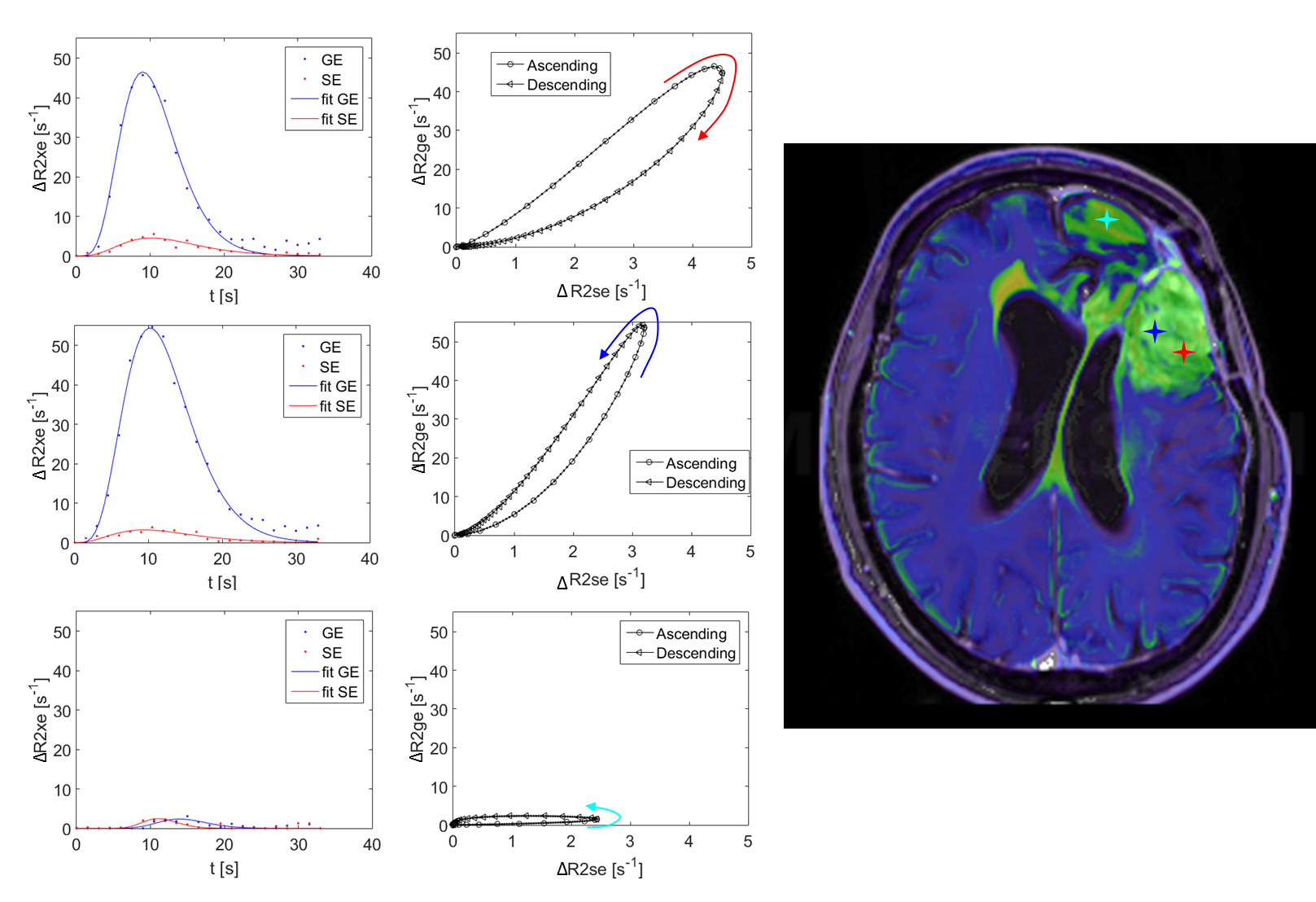

Supplement: S3 Fig — Respective ROIs for three patients are shown on contrast-enhanced T1-weighted MPRAGE images (left hand side) and FLAIR images (right hand side), where the patient in (a) has a glioblastoma (WHO IV) and patient (b) a glioblastoma (WHO IV). Tumor ROIs (in red) were placed at around the contrast-enhancing lesions on the T1-weighted MPRAGE images. Peritumoral edema ROIs (in pink) were placed in representative FLAIR-hyperintense areas neighboring the contrast-enhancing tumor lesions. Peritumoral non-edematous tissue ROIs (in blue) were placed in close vicinity to the contrast-enhancing lesions in a brain area that did show neither contrast-enhancing lesions nor FLAIR-hyperintense lesions. The cNAB ROIs (in yellow) correspond to the mirrored tumor ROI on the contralateral side. (TIF) [file pone.0220939.s005.tif]

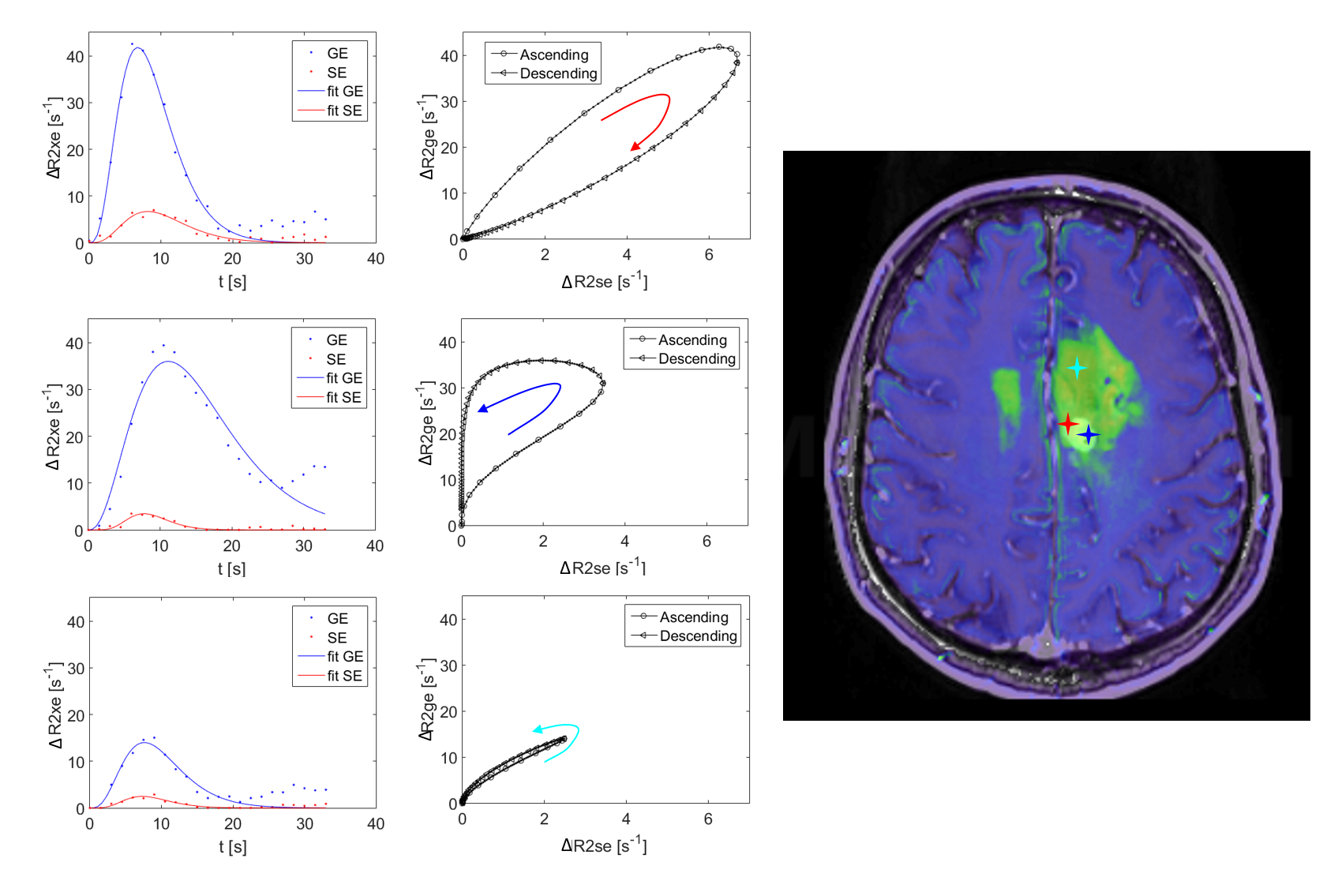

Supplement: S4 Fig — The left column shows relaxation rate time curves taken from three different voxels that are marked with crosses (from top to bottom: red, blue, cyan) in the T1-weighted MPRAGE image with overlaid FLAIR image on the right-hand side. When visualized in a time-parameterized plot, the pairwise GE and SE data points form a vortex curve of a certain shape and in either a clockwise or a counterclockwise direction, see the respective diagrams in the middle column. (TIF) [file pone.0220939.s006.tif]

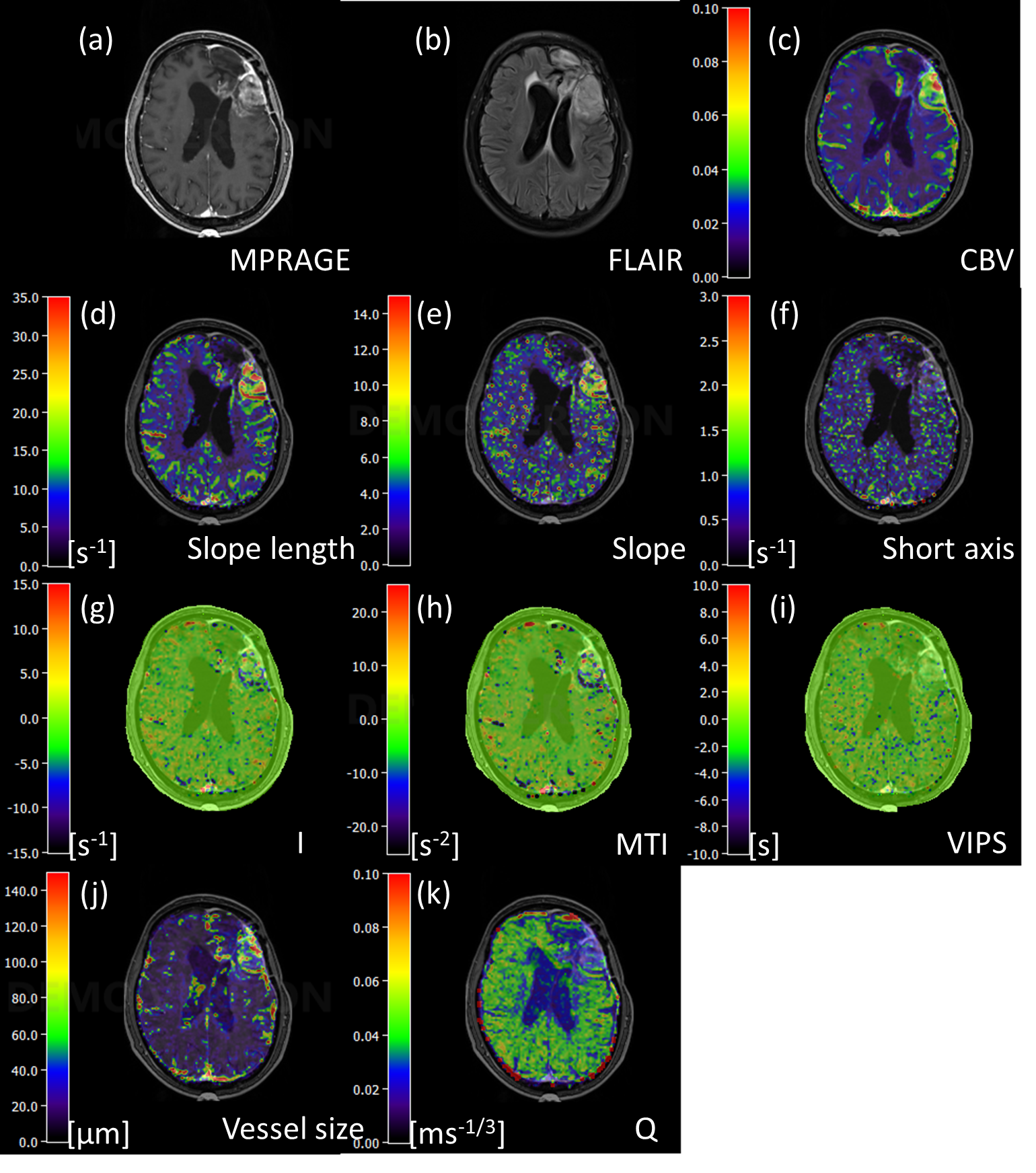

Supplement: S5 Fig — The left column shows relaxation rate time curves taken from three different voxels that are marked with crosses (from top to bottom: red, blue, cyan) in the T1-weighted MPRAGE image with overlaid FLAIR image on the right-hand side. When visualized in a time-parameterized plot, the pairwise GE and SE data points form a vortex curve of a certain shape and in either a clockwise or a counterclockwise direction, see the respective diagrams in the middle column. (TIF) [file pone.0220939.s007.tif]

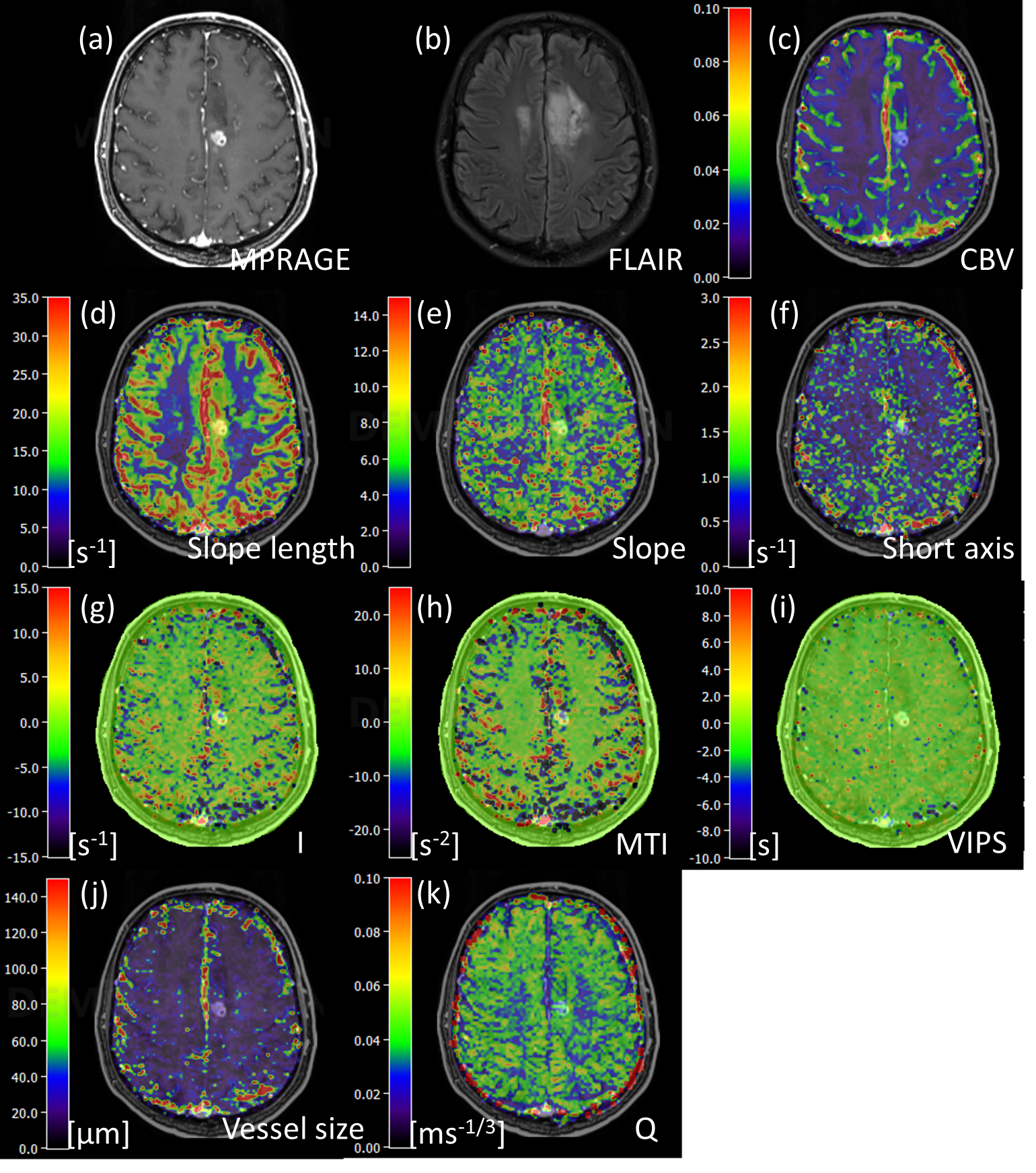

Supplement: S6 Fig — The left column shows relaxation rate time curves taken from three different voxels that are marked with crosses (from top to bottom: red, blue, cyan) in the T1-weighted MPRAGE image with overlaid FLAIR image on the right-hand side. When visualized in a time-parameterized plot, the pairwise GE and SE data points form a vortex curve of a certain shape and in either a clockwise or a counterclockwise direction, see the respective diagrams in the middle column. (TIF) [file pone.0220939.s008.tif]

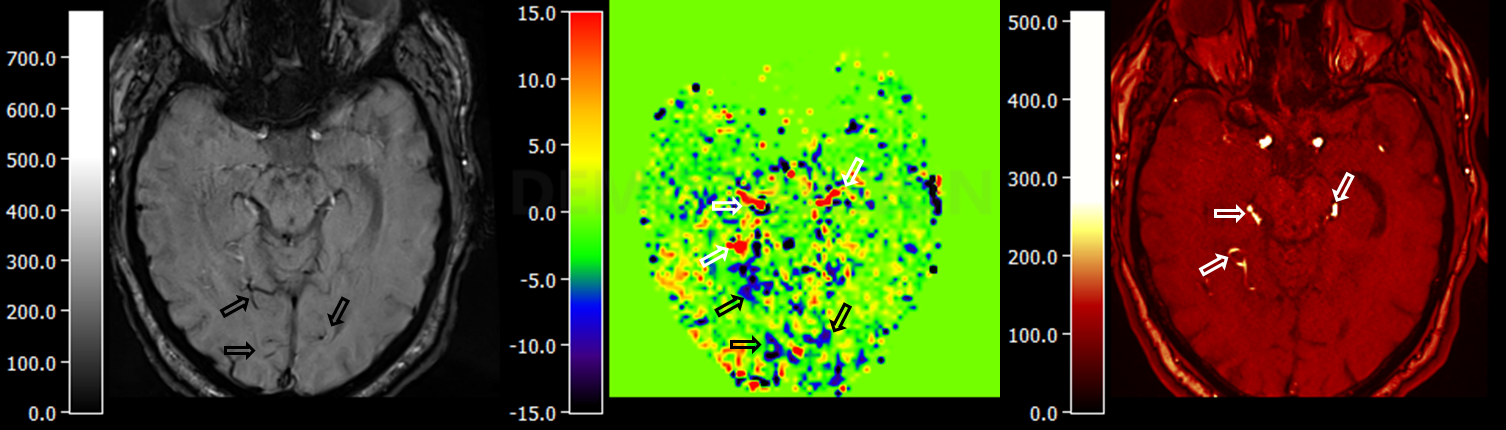

Supplement: S7 Fig — (a) Contrast-enhanced T1-weighted MPRAGE image. (b) FLAIR image. (c) rCBV. (d) VAI slope length. (e) VAI short axis length. (f) VAI slope. (g) VAI distance map. (h) VAI MTI. (i) VAI VIPS. (j) VAI vessel size. (k) VAI Q. For further details, please see main text. (TIF) [file pone.0220939.s009.tif]

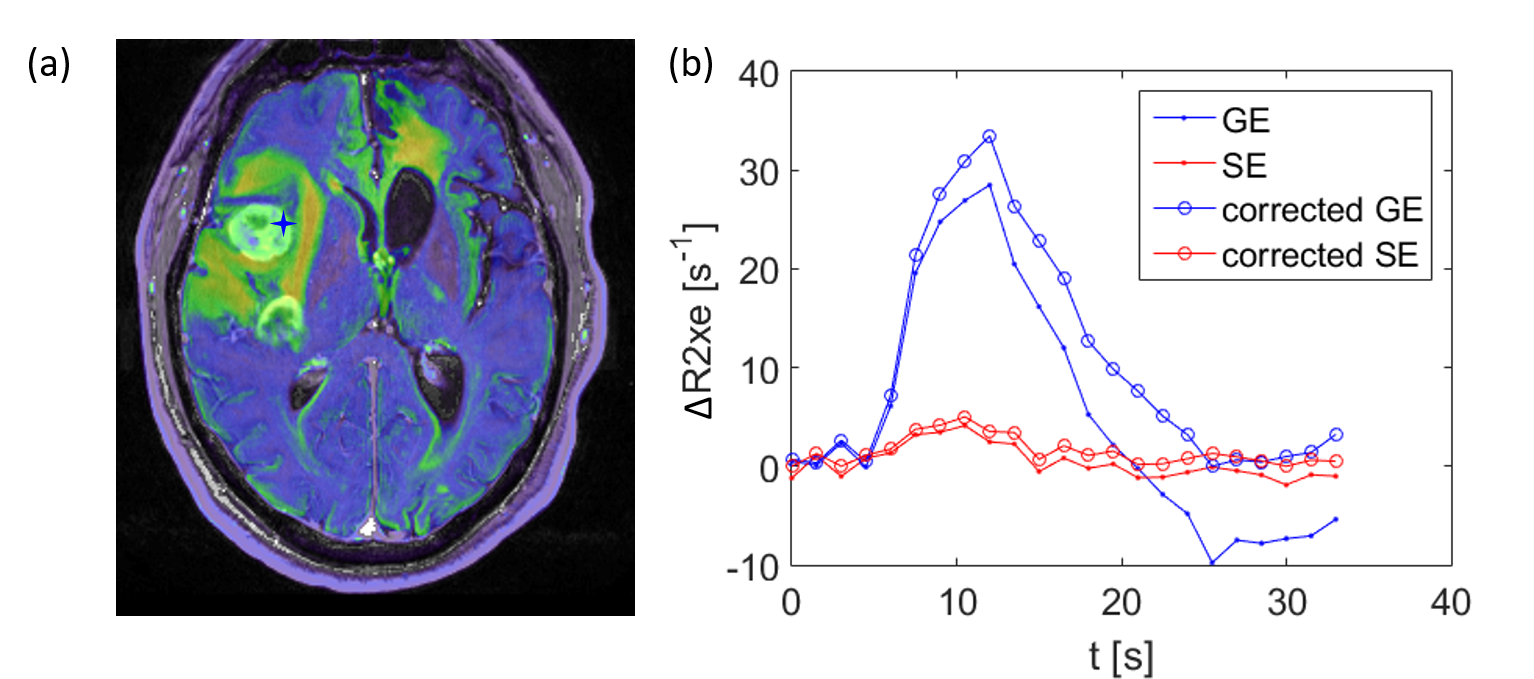

Supplement: S8 Fig — (a) Contrast-enhanced T1-weighted MPRAGE image. (b) FLAIR image. (c) rCBV. (d) VAI slope length. (e) VAI short axis length. (f) VAI slope. (g) VAI distance map. (h) VAI MTI. (i) VAI VIPS. (j) VAI vessel size. (k) VAI Q. For further details, please see main text. (TIF) [file pone.0220939.s010.tif]

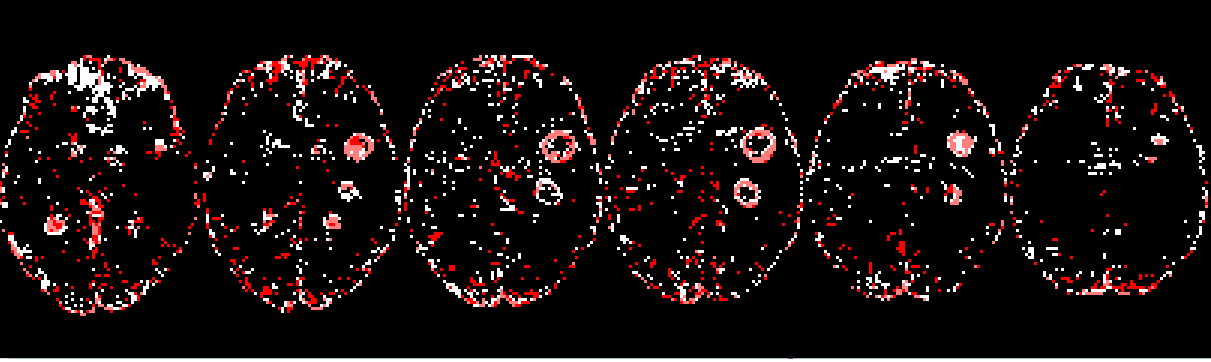

Supplement: S9 Fig — Extreme colors in VAI maps are usually linked to large arteries and veins: intraparenchymal large veins are easily identified on the SWI image (marked with a black arrow; left hand side image) and correspond to large negative values on the distance map (I) (middle image) close to -15s-1. Extreme positive values on the distance map, however, typically correspond to large arteries, e.g. branches of the posterior cerebral artery that were identified on the TOF image (right-hand side of the image) of the same axial slice location (white arrows): corresponding values in the distance map are close to +15s-1. (TIF) [file pone.0220939.s011.tif]
